# Celebration of the Belgian Veterinary Federation in Honor of Professor Alph. Degive, State Director of the School of Veterinary Medicine

**Published:** 1896-06

**Authors:** 


Celebration of the Belgian Veterinary Federaton in Honor of Professor Alph. Degive, State Director of the School of Veterinary Medicine. On December 15, 1895, there was a grand demonstration in honor of Professor Degive. Ever since the elevation to the Presidency of the Royal Academy of Medicine, an event without precedent in the history of the veterinary profession, there had been an unanimous desire among Belgian practitioners to celebrate this event by an official demonstration. The enthusiasm was increased by another event of which the veterinary profession should feel proud, namely, his promotion to the rank of Officier in the Order of Leopold. With one voice the federation decided to celebrate this double distinction with all the *éclat* fitting the occasion, unveiling a bust to his honor, and giving him a banquet. The subscription list was almost completed on the spot, and the whole Belgian corps of veterinarians supported the movement initiated by the federation. The ceremony of unveiling the bust took place in the great auditorium of the school in Brussels, in the presence of a vast concourse of practitioners. A platform upon which the bust had been placed was profusely adorned with flowers and plants, and seats upon it were reserved for Professor Degive, the Bureau of the Federation, and invited guests. At noon exactly the *hero* of the occasion was introduced by the president of the federation amid great applause. M. Bronwier, an honorary member of the same, and member of the Chamber of Representatives, then delivered an eloquent address, in which he reviewed the great services of Professor Degive, and the great debt which the profession in general, as well as Belgium, owed to him. At the close of the address, amid thunderous applause, he unveiled the bust, which is a striking resemblance of Professor Degive. After the applause had subsided, M. Conreur spoke in the name of the students of the Ecole de Cureghem, emphasizing the debt which the students of veterinary medicine owed to the revered teacher. After the platform had been almost deluged with flowers, Professor Degive arose, and, in a voice full of emotion, delivered an eloquent address. He referred feelingly to the great honor which the medical profession had paid to veterinary science in elevating him to the Presidency of the Royal Academy of Medicine; he thanked his co-workers fervently for the great honor which they were paying to him, and closed with an eloquent peroration on the ideals which should ever inspire the veterinarian to new effort and enthusiastic service. After this ceremony was over a banquet was held at the Hôtel Métropole, at which M. Bronwier presided. After drinking the health of the King, and then to Professor Degive, M. Bronwier made a short speech. Several toasts were then drunk, which were responded to by Professor Degive, M. Demarbaix, Professor at the University of Louvain; Dr. Jacobs, Professor Labo, and others.—*Annales de Médecine Vétérinaire*, January and February, 1896.

